# Circulating microRNAs Profile in Patients With Transthyretin Variant Amyloidosis

**DOI:** 10.3389/fnmol.2020.00102

**Published:** 2020-06-23

**Authors:** Gian Luca Vita, M’Hammed Aguennouz, Francesca Polito, Rosaria Oteri, Massimo Russo, Luca Gentile, Cristina Barbagallo, Marco Ragusa, Carmelo Rodolico, Rosa Maria Di Giorgio, Antonio Toscano, Giuseppe Vita, Anna Mazzeo

**Affiliations:** ^1^Nemo Sud Clinical Centre for Neuromuscular Disorders, Messina, Italy; ^2^Unit of Neurology and Neuromuscular Diseases, Department of Clinical and Experimental Medicine, University of Messina, Messina, Italy; ^3^Mohammed VI University of Health Sciences, Casablanca, Morocco; ^4^Molecular, Genome and Complex Systems BioMedicine Unit, Department of Biomedical and Biotechnological Sciences, University of Catania, Catania, Italy; ^5^Oasi Research Institute IRCCS, Troina, Italy

**Keywords:** transthyretin, amyloidosis, microRNAs, miR-150-5p, CREB, BDNF, NGF

## Abstract

Transthyretin variant amyloidosis (ATTRv) is a rare autosomal dominant disease characterized by the accumulation of amyloid in many organs, mostly causing a sensory-motor neuropathy, cardiomyopathy, and dysautonomia. The aim of the study was to report microRNAs (miRNAs) expression profile identified in the blood of ATTRv patients. Ten ATTRv patients, 10 asymptomatic carriers of transthyretin variant (TTRv), 10 patients with Charcot-Marie-Tooth (CMT) disease, and 10 healthy controls were studied. Human Schwann cells cultures were used to study the regulatory effects of miR-150-5p on the expression of cAMP response element-binding protein (CREB), brain-derived neurotrophic factor (BDNF), and nerve growth factor (NGF). ATTRv patients had 33 miRNAs up-regulated and 48 down-regulated versus healthy controls; 9 miRNAs were up-regulated and 30 down-regulated versus CMT patients; 19 miRNAs were up-regulated and 38 down-regulated versus asymptomatic TTRv carriers. Twelve out of the 19 upregulated miRNAs had a fold increase higher than 100. The validation experiment indicated miR-150-5p as a valuable biomarker to differentiate ATTRv patients from asymptomatic TTRv carriers (AUC: 0.9728; *p* < 0.0001). Schwann cells culture model demonstrated that miR-150-5p is a powerful negative regulator of CREB, BDNF, and NGF genes. Identification of deregulated miRNAs can help in understanding the complex pathomechamism underlying the development of ATTRv and related multisystemic pathology. Further investigations are needed on the role of circulating miR-150-5p to predict the shift of TTRv carriers from an asymptomatic status to symptoms appearance.

## Introduction

Transthyretin (TTR) variant amyloidosis (ATTRv) is an autosomal dominant disease characterized by the formation and storage of amyloid aggregates in many organs, mostly causing an axonal sensory-motor neuropathy, cardiomyopathy, gastrointestinal dysfunction, and dysautonomia. More than 120 TTR variants (TTRv) are known with several thousand cases worldwide, and organ involvement and clinical symptoms widely depend on the type of mutation (Adams et al., 2017). A staging system classifies patients into three stages based on severity of symptoms and the extent of disease progression, being stage 1 for symptomatic subjects with unimpaired walking, stage 2 for need of mono- or bilateral walking assistance, and stage 3 for patients relying on a wheelchair to move around. Stage 0 is an asymptomatic stage for subjects who have a TTRv but do not show yet any symptoms of the disease (Adams et al., 2016). Based on phase 3 clinical trials, the effectiveness of current treatment options is limited to stage 1 and 2 ATTRv patients (Vita et al., 2019), so that regular follow-ups are done in asymptomatic carriers to monitor the appearance of the first symptoms and to start treatment (Obici et al., 2016). Several neurophysiological and cardiological markers of early damage have been recently proposed (Jonker et al., 2018; Luigetti et al., 2018; Zouari et al., 2019). At the moment, no biomarker is available to document the progression from an asymptomatic to symptomatic status.

MicroRNAs (miRNAs) are small (20–25 nucleotides), evolutionary conserved, non-coding RNAs which negatively regulate gene expression by binding to the 3’-untranslated region of target mRNAs in a sequence-specific manner, leading to either mRNA degradation or translational repression. They can also positively regulate gene expression by repression of several negative regulators (Kong et al., 2012). They are expressed specifically in different tissues but circulating miRNAs have been recognized in human plasma and serum, since they are passively leaked or actively transported from cells. More specifically, miRNAs play key roles in vital biological processes such as cell division and death, metabolism, intracellular signaling, immunity, and cell movement (Baltimore et al., 2008; Png et al., 2011; Rayner et al., 2011; Ng et al., 2012). In the last years, circulating miRNAs emerged as an interesting new class of biomarkers, being easily measured using common laboratory techniques, with potential clinical relevance for diagnosis and prognosis, to predict responders from non-responders to a given treatment, or even as therapeutic targets (Huang et al., 2017; Molasy et al., 2017; Li et al., 2019; Fattahi et al., 2020). We performed a pilot study to determine whether circulating miRNAs could be identified in the blood of ATTRv patients and if so, whether they are linked to disease stage.

## Materials and Methods

Blood samples were obtained from 10 ATTRv patients (stage 1 or 2), 10 asymptomatic carriers of TTRv, 5 patients with axonal-type Charcot-Marie-Tooth (CMT) disease type 2 and 5 patients with CMT type 1A as pathological controls, and 10 healthy controls (HC) (Table 1). The ATTRv patients and the asymptomatic subjects carried the following TTR mutations: Glu89Gln (n. 4 and n. 4, respectively), Phe64Leu (n. 4 and n. 4), Thr49Ala (n. 2 and n. 2) (Mazzeo et al., 2015). All 10 symptomatic patients presented neuropathy, 5/10 had cardiopathy, and 7/10 had dysautonomia. All were on treatment with tafamidis. Patients had no concomitant major disease, such as diabetes, hypertension, ischemic heart disease, neoplasm, cerebrovascular disease, etc. Local ethics committee approved the study and all subjects gave written informed consent.

**TABLE 1:** Populations studied.

	ATTRv patients	Asymptomatic TTRv carriers	CMT patients	Healthy controls
**Discovery set**				
No.	10	10	10	10
Male/female	5/5	4/6	6/4	5/5
Age (years)	52.8 ± 6.3	44.0 ± 7.0	43.5 ± 18.7	48.0 ± 7.5
Disease duration (years)	6.7 ± 4.0	NA	17.0 ± 14.8	NA
**Validation set**				
No.	24	23	–	–
Male/female	15/9	10/13	–	–
Age (years)	63.7 ± 12.1	49.1 ± 8.3	–	–
Disease duration (years)	7.7 ± 4.9	NA	–	–

*Mean ± SD; NA, not applicable.*

Blood samples were withdrawn by venipuncture into BD Vacutainer tubes with a gel separating serum from blood cells. The samples were centrifuged at 3500 RPM for 15 min at 4°C. Supernatant was isolated and centrifuged again to remove circulating cells or debris. Aliquots of serum were stored at −80°C, until analysis.

### RNA Isolation

Eight hundred microliter serum samples were used to extract total RNA using Qiagen miRNeasy Mini Kit (Qiagen, GmbH, Hilden, Germany), according to Qiagen Supplementary Procedure for the purification of RNA, including small RNAs. Obtained RNA was eluted in 200 μl RNAse-free water and then precipitated adding 20 μg glycogen, 0.1 volumes 3 M sodium acetate, and 2.5 volumes ice cold 100% ethanol. Following overnight incubation at –80°C, RNA was centrifuged and twice washed in ice cold 75% ethanol and resuspended in 7 μl RNAse-free water. RNA was quantified by Nanodrop.

### Circulating miRNA Profiling

Serum miRNA expression profile was done by nCounter Human v3 miRNA Expression Assay Kit (NanoString Technologies; Seattle, WA, United States) in an nCounter FLEX (Prep Station and Digital Analyzer) (NanoString Technologies), according to manufacturer procedures. Three microliter, containing about 100 ng of total RNA, were utilized for sample preparation. Data analysis was made through nSolver 2.6 software (NanoString Technologies). MiRNAs used as endogenous controls were chosen through global median normalization method: we computed Pearson correlation between the count means for each lane and the counts of each miRNA, identifying those miRNAs whose expression was closer to the count mean of the cartridge (miR-23a-3p, miR-1285-5p, miR-451a) (Di Pietro et al., 2018).

Single miRNA qRT validation analysis was performed on 20 ng of total RNA by using single TaqMan^®^ MicroRNA Assays (Applied Biosystems) in a 7300 Real time PCR instrument (Thermo Fisher Scientific, Waltham, MA, United States), according to the manufacturer’s instructions. Expression fold changes were calculated by the 2^–ΔΔCT^ method by using RNU6 as reference genes. All assays were performed in triplicate.

### Gene Ontology and Pathway Enrichment Analysis

To investigate the effects of the deregulation of miRNAs, a gene ontology (GO) analysis was performed following two parallel approaches. Experimentally validated targets of upregulated miRNAs were retrieved from TarBase v7.0^1^. The resulting list of mRNA targets was used as input for the TopGO v2.24.0 package, which analyzes the GO database. Moreover, upregulated miRNAs were submitted to the tool DIANA mirPath v3.0^2^, which performed a pathway enrichment analysis according to the Kyoto Encyclopedia of Genes and Genomes (KEGG). DIANA mirPath was performed on experimentally validated miRNA targets retrieved from Tarbase v7.0 by appling FDR correction (*p* < 0.05, MicroT < 0.8) and Fisher’s Exact Test (Hypergeometric Distribution).

### Cell Culture

Human primary Schwann cells (SCs) (ABM Good, Richmond, Canada) were cultured in Prigrow X series medium (ABM Good) containing 10% fetal bovine serum (Gibco, Gaithersburg, MD, United States), 100 μg/ml streptomycin, and 100 IU/ml penicillin (Sigma, St. Louis, MO, United States) at 37°C in a 5% CO_2_ humidified atmosphere. The cells were subcultured every 2–3 days.

### miRNA Transfections

miR-150-5p mimic/inhibitor (ID MC10070/MH10070; Thermo Fisher Scientific) were transfected into human primary SCs using siPORT Lipid Transfection Reagent (Thermo Fisher Scientific) according to the manufacturer’s procedure. Cells were transfected with 50 nmol of oligonucleotide per well (0.5 × 10^6^ cells). Transfected cells were assayed 24 and 48 h after the transfection.

### Western Blot Analysis

SCs samples were processed in lysis buffer (25 mM Tris/HCL, pH 7.4, 1.0 mM EGTA, 1.0 mM ethylen diamine tetraacetic acid (EDTA), protease, and phosphatase inhibitors) and total proteins concentration was determined using the Bio-Rad protein assay kit (Bio-Rad, Richmond, CA, United States). Thirty micrograms of proteins were resolved by SDS-PAGE, separated by electrophoresis, and blotted onto PVDF membrane (Amersham Bioscience, Amersham, United Kingdom). Membranes were incubated with specific antibodies against cAMP response element-binding protein (CREB) (1:200; catalog #sc-240; Santa Cruz Biotechnology, CA, United States), brain-derived neurotrophic factor (BDNF) (1:200; catalog #sc-65514; Santa Cruz Biotechnology), or nerve growth factor (NGF) (1:500; catalog #MA5-32067; Invitrogen, Waltham, MA, United States). Equal loading of protein was assessed on stripped blots by immunodetection of β-actin (1:500; Abcam, Cambridge, MA, United States). For all primary antibodies, a peroxidase-conjugated goat anti-rabbit immunoglobulin G secondary antibody was used at concentration of 1:10,000 (catalog #G-21234; Pierce, Chester, United Kingdom). Signals were detected using Amersham ECL Plus Western Blotting Detection Reagents (Amersham Bioscience). Computer-assisted densitometry (UN-SCAN-IT gel version 6.1; Silk Scientific, Inc., Orem, UT, United States) was used to perform semi-quantitative analysis of protein expression detected by immunoblotting. Different times of exposure were used for each blot. β-actin signal was used to normalize protein levels. Integrated density values were expressed as a percentage of densitometric levels using arbitrary densitometric units (Vita et al., 2018).

### Real Time-Quantitative Polymerase Chain Reaction (RT-qPCR)

Total RNA was isolated with Trizol Reagent (Invitrogen) according to the manufacturer’s protocol. Five micrograms of RNA from each sample were reversely transcribed using High-Capacity cDNA Archive Kit (Applied Biosystems, Foster City, CA, United States). Generated cDNA was used as a template for RT-qPCR analysis. Briefly, for each reaction, 4 μl of cDNA in a total volume of 50 μl were used. 7300 Sequence Detection System apparatus (Applied Biosystems) was managed to quantitatively compare the mRNA levels; 20X target primer and probe (BDNF: HS02718934; NGF: HS00171458; CREB: HS00231713) were processed, and human β-actin (Cod.4326315E) was used as a house-keeping gene (Thermo Fisher Scientific). RT-qPCR was done in duplicate with 2 × TaqMan Universal PCR Master Mix. The thermal cycling conditions were as following: 10′ at 25°C, 120′ at 37°C, and then hold at 4°C. The comparative cycle threshold (Ct) procedure (Applied Biosystems) was used to analyze the data by generating relative values of target cDNA level. Relative quantification (RQ) was expressed as fold change over control, and calculated by the ΔΔCt method, with control samples as calibrators.

### Statistical Analysis

For NanoString analysis, fold change (FC) expression changes between two groups were calculated by using nSolver v2.5 (NanoString Technologies) ratio data, based on normalized count data. *p* values between two groups were generated using a two-tailed t-test. Statistical analysis was done through Significance of Microarrays Analysis^3^, using a *p* value based on 100 permutations; imputation engine: K-nearest neighbors (10 neighbors); false discovery rate (FDR) <0.05. Quantitative RT-qPCR microRNA expression was assessed using SDS v2.4 software (Thermo Fisher Scientific) and analyzed using GraphPad Prism version 8.3.0. Results are expressed as mean ± standard deviation (SD). Receiver operating characteristic (ROC) analysis was used to assess the performance of miRNA as a binary expression status in symptomatic and asymptomatic carriers of TTRv. The area under the ROC curve (AUC) was also determined. Statistical multiple comparison between groups was performed by Kruskal-Wallis non-parametric ANOVA test. Comparison between groups was performed by Mann-Whitney test for unpaired non-parametric data. A level of significance of *p* < 0.05 was considered.

## Results

Among up to 800 biologically relevant miRNAs, 33 miRNAs were found significantly up-regulated and 48 down-regulated in the serum of ATTRv patients versus HC. Nine miRNAs were significantly up-regulated and 30 down-regulated in ATTRv patients versus CMT patients. With the aim to find differences between ATTRv patients versus asymptomatic carriers of TTRv, expression analysis led to identify 19 miRNAs significantly up-regulated and 38 miRNAs down-regulated in patients versus asymptomatic carriers. Twelve out of the 19 upregulated miRNAs had a fold increase higher than 100 (Table 2; GEO Series accession number GSE149665).

**TABLE 2:** Differentially expressed miRNAs.

	ATTRv patients vs HC		ATTRv patients vs CMT patients		ATTRv patients vs TTRv carriers	
			
	Fold change	*p*-value	Fold change	*p*-value	Fold change	*p*-value
hsa-let-7d-5p	2.61	0.003	–	–	1.38	0.02
hsa-let-7i-5p	2.21	0.002	–	–	–50.13	2.51E-09
hsa-miR-10a-5p	–	–	–1.43	0.01	–	–
hsa-miR-15a-5p	–	–	–	–	–1.54	0.02
hsa-miR-19a-3p	–2.43	0.0006	–	–	–	–
hsa-miR-20a-5p + hsa-miR-20b-5p	3.09	0.0006	–	–	–	–
hsa-miR-21-5p	3.5	0.001	2.34	0.006	77.57	0.0001
hsa-miR-22-3p	2.28	0.003	–	–	–	–
hsa-miR-25-3p	–2.03	0.005	–	–	–	–
hsa-miR-26b-5p	2.6	0.001	–	–	2.26	0.0008
hsa-miR-28-5p	–	–	2.68	0.0001	–	–
hsa-miR-30a-5p	–2.38	0.01	–	–	–	–
hsa-miR-33a-5p	–2.42	0.0005	–	–	–	–
hsa-miR-34a-5p	1.74	0.03	–	–	106.74	0.00008
hsa-miR-93-5p	–2.31	0.001	–	–	–	–
hsa-miR-96-5p	1.98	0.03	–	–	–75.21	0.00000253
hsa-miR-107	1.84	0.0006	–	–	104.9	1.69E-08
hsa-miR-124-3p	–2.37	0.002	–	–	–	–
hsa-miR-125a-3p	2.79	0.0006	–	–	–	–
hsa-miR-126-3p	–	–	–3.32	0.001	–	–
hsa-miR-132-3p	–2.09	0.01	–	–	–	–
hsa-miR-133b	–2.22	0.001	–	–	–	–
hsa-miR-138-5p	–	–	–1.85	0.01	–	–
hsa-miR-141-3p	–2.54	0.0004	–	–	–	–
hsa-miR-142-3p	–	–	–	–	–2.25	0.003
hsa-miR-144-3p	–2.15	0.001	–7.97	0.0001	–	–
hsa-miR-146a-5p	1.71	0.01	–	–	–50.56	0.000000411
hsa-miR-146b-5p	–	–	–2.05	0.006	–	–
hsa-miR-147a	–2.61	0.001	–	–	–	–
hsa-miR-150-5p	1.6	0.02	1.7	0.03	109.23	0.00000111
hsa-miR-181a-5p	–	–	–1.61	0.003	–	–
hsa-miR-181b-2-3p	–	–	–2.23	0.0006	–	–
hsa-miR-184	–	–	–	–	–50.41	0.00000111
hsa-miR-188-5p	–1.78	0.001	–1.87	0.001	–	–
hsa-miR-190a-3p	–1.91	0.004	–	–	–	–
hsa-miR-196a-5p	–	–	–1.77	0.001	–	–
hsa-miR-199a-3p + hsa-miR-199b-3p	–2.68	0.008	–	–	–	–
hsa-miR-200a-3p	–	–	–1.69	0.0003	–	–
hsa-miR-206	1.58	0.008	–	–	–50.33	0.00000183
hsa-miR-208b-3p	2	0.0004	–	–	68.44	0.0000031
hsa-miR-210-3p	–2.15	0.003	–	–	–	–
hsa-miR-215-5p	2.13	0.006	–	–	101.96	0.000000688
hsa-miR-216a-5p	–1.74	0.002	–	–	–	–
hsa-miR-216b-5p	–	–	–1.92	0.0005	–	–
hsa-miR-219b-3p	–2.43	0.0002	–	–	–53.14	0.00000127
hsa-miR-223-3p	1.98	0.002	–	–	–	–
hsa-miR-224-5p	–	–	–1.37	0.006	–	–
hsa-miR-302e	–2.18	0.003	–	–	–50.21	0.00000535
hsa-miR-320a	–2.14	0.003	–	–	–	–
hsa-miR-320e	–2.33	0.02	–	–	–	–
hsa-miR-323b-3p	–5.09	0.0008	–2.1	0.0002	–70.23	0.000002
hsa-miR-328-5p	2.52	0.002	–	–	–	–
hsa-miR-335-5p	2.04	0.009	–	–	–	–
hsa-miR-339-5p	–	–	–1.8	0.0005	–	–
hsa-miR-363-5p	1.81	0.04	–	–	65.44	0.0001
hsa-miR-376a-3p	–	–	–1.92	0.04	–	–
hsa-miR-378d	–	–	–1.95	0.01	–	–
hsa-miR-378i	2.2	0.0001	–	–	–70.11	0.000006
hsa-miR-411-5p	–2.14	0.02	–	–	–2.01	0.004
hsa-miR-422a	–2.42	0.0002	–	–	–	–
hsa-miR-450a-2-3p	–2.24	0.0005	–	–	–50.19	0.00001
hsa-miR-452-5p	–2.27	0.002	–	–	–	–
hsa-miR-495-3p	1.79	0.03	–	–	–50.28	0.00006
hsa-miR-497-5p	2.27	0.01	–	–	51.81	0.00000106
hsa-miR-514b-5p	–	–	–	–	–52.12	0.00000956
hsa-miR-518c-3p	–3.17	0.001	–	–	–	–
hsa-miR-518d-3p	–2.87	0.0002	–	–	–	–
hsa-miR-526a + hsa-miR-518c-5p + hsa-miR-518d-5p	–	–	–	–	–71.41	0.00000121
hsa-miR-548ad-3p	–1.74	0.0008	–2.06	0.0009	–71.2	0.000000891
hsa-miR-548ah-5p	–2.04	0.01	–	–	–51.94	0.00006
hsa-miR-548ar-3p	–1.76	0.0008	–	–	–50.16	0.00007
hsa-miR-566	–	–	–	–	–70.31	0.000000465
hsa-miR-575	2.02	0.01	3.24	0.001	108.57	0.0001
hsa-miR-584-5p	–	–	2.62	0.008	–	–
hsa-miR-585-3p	1.76	0.01	–	–	–1.7	0.0003
hsa-miR-587	2.44	0.006	–	–	102.39	0.00003
hsa-miR-593-3p	–	–	–1.89	0.0001	–	–
hsa-miR-598-3p	–2.02	0.002	–	–	–	–
hsa-miR-603	2.49	0.03	–	–	–	–
hsa-miR-607	–2.11	0.01	–	–	–	–
hsa-miR-612	–2.06	0.0002	–1.71	0.0001	–	–
hsa-miR-637	–2	0.0006	–	–	–	–
hsa-miR-663a	–	–	–	–	–72.96	0.00000086
hsa-miR-758-5p	–2.72	0.01	–	–	–	–
hsa-miR-802	–	–	–	–	–71.52	0.00000551
hsa-miR-873-3p	–2.44	0.005	–1.43	0.0004	–71	8.67E-08
hsa-miR-876-3p	1.53	0.009	–	–	121.29	0.00001
hsa-miR-877-5p	–2.03	0.0005	–	–	117.34	0.0001
hsa-miR-887-5p	8.68	0.0002	–	–	–	–
hsa-miR-889-3p	1.75	0.001	2.11	0.0002	112.77	9.41E-14
hsa-miR-891b	–1.51	0.001	–	–	66.38	0.000000747
hsa-miR-922	–2.21	0.03	–	–	–57.32	0.00000241
hsa-miR-933	–	–	–1.51	0.003	–	–
hsa-miR-936	–2.09	0.0001	–	–	–50.35	0.000000841
hsa-miR-941	–	–	–1.85	0.002	–	–
hsa-miR-1185-1-3p	–2.1	0.005	–	–	–	–
hsa-miR-1185-2-3p	–2.8	0.0001	–	–	–2.32	0.003
hsa-miR-1261	–2.36	0.01	–	–	–	–
hsa-miR-1268b	–2.21	0.0005	–	–	–53.52	0.000000168
hsa-miR-1277-3p	2.75	0.01	–	–	–	–
hsa-miR-1279	3.07	0.006	–	–	119.18	0.00001
hsa-miR-1285-3p	–	–	–	–	–2.18	0.003
hsa-miR-1287-5p	1.8	0.009	2.53	0.002	104.7	0.000000611
hsa-miR-1296-3p	5.93	0.001	–	–	–61.1	4.61E-17
hsa-miR-1304-3p	–	–	–2.05	0.0003	–	–
hsa-miR-1304-5p	–2.28	0.001	2.03	0.01	102.09	0.0005
hsa-miR-1322	–2.37	0.006	–	–	–	–
hsa-miR-1827	–	–	1.73	0.03	–1.96	0.001
hsa-miR-1972	–	–	–	–	–52.55	9.26E-08
hsa-miR-2117	–	–	–2.1	0.03	–	–
hsa-miR-3168	–	–	–	–	–73.46	7.01E-08
hsa-miR-3614-5p	–	–	–	–	–1.81	0.0004
hsa-miR-3615	–	–	–1.86	0.002	–	–
hsa-miR-3928-3p	–	–	–1.59	0.0004	–	–
hsa-miR-4421	–	–	–1.5	0.02	–59.22	0.00000135
hsa-miR-4454 + hsa-miR-7975	–2.17	0.005	–	–	–2.2	0.04
hsa-miR-4516	–	–	–	–	–1.67	0.01
hsa-miR-4536-5p	–2.03	0.001	–	–	–	–
hsa-miR-4787-5p	–	–	–	–	–70.66	0.000000199
hsa-miR-5010-3p	–	–	–1.52	0.002	–	–
hsa-miR-6720-3p	–	–	–2.1	0.0002	–	–
hsa-miR-6721-5p	–	–	–1.54	0.0006	–	–

Bioinformatic analysis of upregulated miRNAs found in ATTRv patients vs TTRv carriers revealed that the target genes were involved in a variety of cellular functions. The top significantly related GO terms are listed in Table 3. The KEGG analysis showed the involvement of the upregulated target genes in important pathways such as axon guidance, the mitogen-activated protein kinase (MAPK) cascade, and the forkhead box O (FOXO) family of transcription factors, the last two being involved in proliferation, apoptosis, differentiation, and cell-cycle control (Table 4).

**TABLE 3:** GO analysis of targets of upregulated miRNAs found in ATTRv patients vs TTRv carriers.

Category	Term	Count	*p*-value	FDR
Biological process	GO:0007399∼nervous system development	175	7.89E-12	1.75E-08
	GO:0065008∼regulation of biological quality	174	6.25E-11	6.78E-09
	GO:0023051∼regulation of signaling	176	4.12E-12	6.81E-09
	GO:0007167∼enzyme linked receptor protein signaling pathway	105	2.33E-11	4.26E-08
	GO:0010604∼positive regulation of macromolecule metabolic process	198	2.72E-11	5.34E-08
	GO:0010646∼regulation of cell communication	148	2.22E-09	2.23E-09
Molecular function	GO:0070491∼repressing transcription factor binding	124	2.54E-11	6.57E-09
	GO:0061659∼ubiquitin-like protein ligase activity	118	3.12E-10	3.21E-09
	GO:0019899∼enzyme binding	122	0.00000179	1.18E-09
	GO:0019904∼protein domain specific binding	108	0.00000358	1.26E-08
	GO:0005057∼receptor signaling protein activity	48	2.98E-08	0.0000451
	GO:0140096∼catalytic activity, acting on a protein	57	3.01E-08	3.23E-09
Cellular components	GO:0070062∼extracellular exome	178	2.98E-11	2.88E-09
	GO:0044451∼nucleoplasm part	122	0.00000184	1.21E-09
	GO:0044424∼intracellular part	52	0.00000181	1.18E-08
	GO:0005057∼receptor signaling protein activity	68	2.11E-08	0.0000374

**TABLE 4:** KEGG pathway analysis of targets of upregulated miRNAs found in ATTRv patients vs TTRv carriers.

KEGG pathways	Count	*p*-value	FDR
hsa04360: Axon guidance	78	0.0000083	0.0000531
hsa04010: MAPK signaling pathway	58	0.0000287	0.0000019
hsa04068: FoxO signaling pathway	62	0.0000482	0.0000021

**FIGURE 1:**
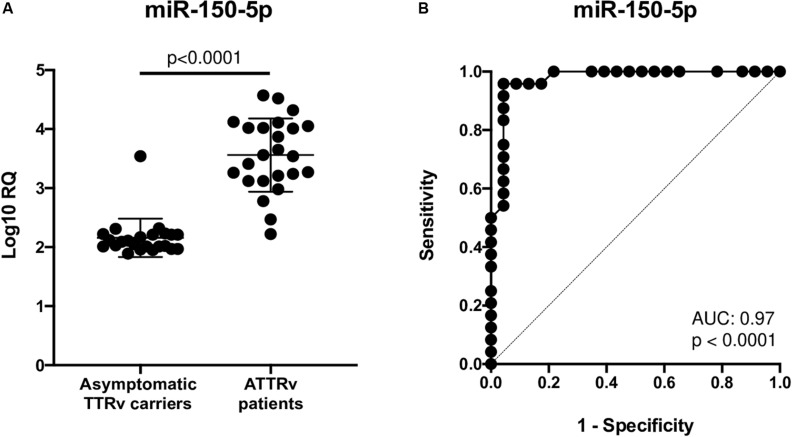
Expression levels of circulating miR-150-5p in 23 asymptomatic TTRv carriers and 24 ATTRv patients **(A)**. Receiver operating characteristic (ROC) curve analysis of miR-150-5p **(B)**. AUC resulted 0.9728 (95% CI: 0.9285–1.000). At the cutoff value of 2.395, sensitivity and specificity were 95.8 and 95.6%, respectively.

After a database search and literature review of the twelve most up-regulated miRNAs in ATTRv patients versus asymptomatic carriers of TTRv, miR-150-5p was selected for further validation. miR-150-5p expression was measured by RT-qPCR in the serum of n. 24 ATTRv patients and n. 23 asymptomatic carriers of TTRv (Table 1). The results revealed that miR-150-5p was significantly up-regulated in patients compared with asymptomatic carriers (Figure 1A and Supplementary Table S1). ROC curve analysis indicated that miR-150-5p was a valuable serum biomarker for differentiating with an AUC of 0.9728 (95% CI: 0.9285–1.000). At the cutoff value of 2.395, sensitivity and specificity were 95.8 and 95.6%, respectively (Figure 1B).

**FIGURE 2:**
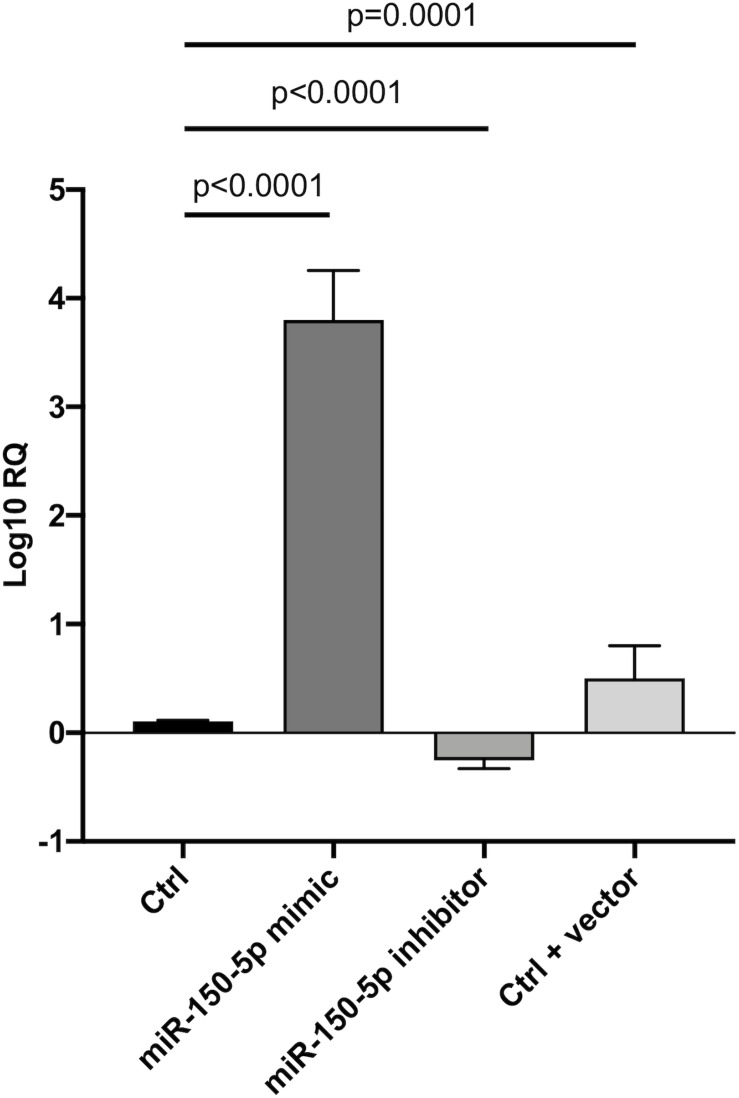
Transfection of miR-150-5p mimic and inhibitor into human primary Schwann cells and its effect on miR-150-5p expression. Non-parametric ANOVA Kruskal-Wallis test *p* < 0.0001.

**FIGURE 3:**
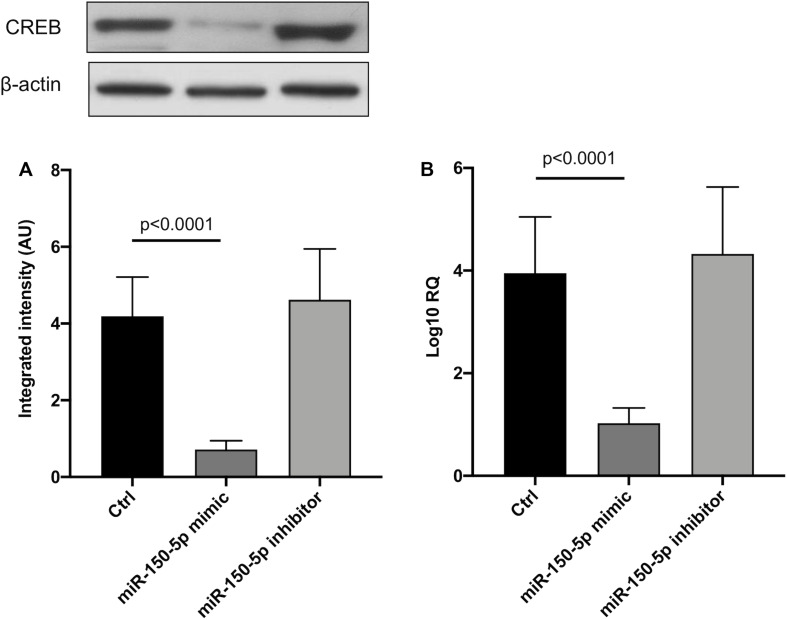
CREB protein **(A)** and mRNA **(B)** levels in transfected human primary Schwann cells. **(A)** Lower panel shows graph with quantitative data; upper panel shows representative autoradiograms. Independent experiments were performed in triplicate. Non-parametric ANOVA Kruskal-Wallis test *p* < 0.0001 in both experiments.

**FIGURE 4:**
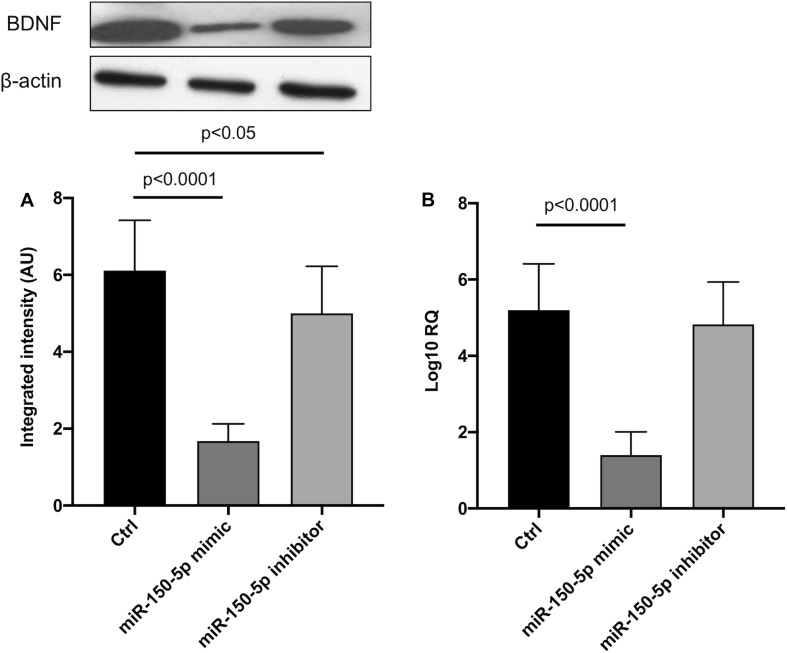
BDNF protein **(A)** and mRNA **(B)** levels in transfected human primary Schwann cells. **(A)** Lower panel shows graph with quantitative data; upper panel shows representative autoradiograms. Independent experiments were performed in triplicate. Non-parametric ANOVA Kruskal-Wallis test *p* < 0.0001 in both experiments.

**FIGURE 5:**
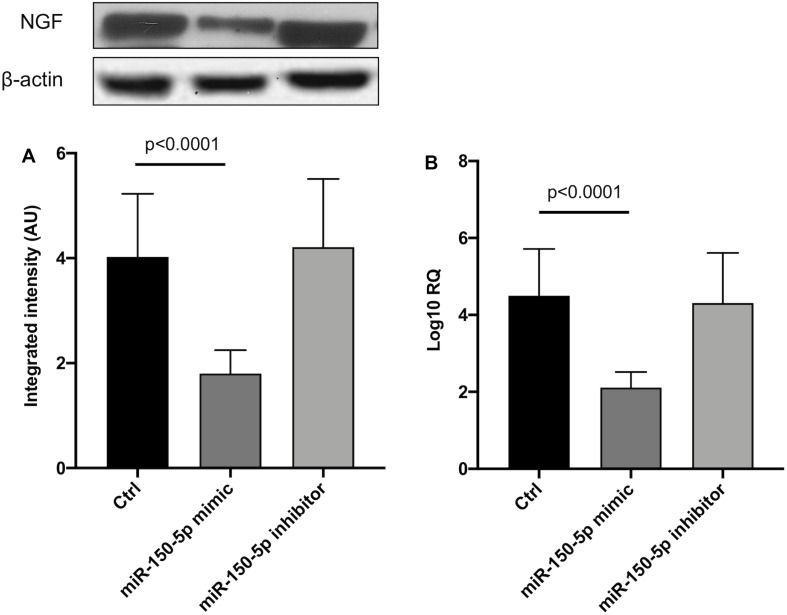
NGF protein **(A)** and mRNA **(B)** levels in transfected human primary Schwann cells. **(A)** Lower panel shows graph with quantitative data; upper panel shows representative autoradiograms. Independent experiments were performed in triplicate. Non-parametric ANOVA Kruskal-Wallis test *p* < 0.0003 and *p* < 0.0005, respectively.

Since in ATTRv mutated transthyretin aggregates and forms amyloid fibrils in target organs, chiefly the peripheral nervous system and the heart, the expression of three regulators of neuronal growth, differentiation, survival and regeneration, such as CREB, BDNF and NGF, which are also involved in cardiac dysfunction, were investigated in human SCs cultures transfected by miR-150-5p mimic/inhibitor. Treatment with miR-150-5p mimic markedly increased the miR-150-5p expression by around 30-fold when compared to control (*p* < 0.0001), whereas treatment with miR-150-5p inhibitor suppressed its expression (*p* < 0.0001) (Figure 2). CREB protein and mRNA levels decreased after transfection of miR-150-5p mimic by almost 6-fold (*p* < 0.0001) and almost 4-fold (*p* < 0.0001), respectively (Figure 3). BDNF protein and mRNA levels were decreased after transfection of miR-150-5p mimic by almost 4-fold (*p* < 0.0001) and almost 4-fold (*p* < 0.0001), respectively (Figure 4). NGF protein and mRNA levels reduced after transfection of miR-150-5p mimic by 2-fold (*p* < 0.0001) and 2-fold (*p* < 0.0001), respectively (Figure 5). Data from SCs culture experiments are listed in Supplementary Table S2.

## Discussion

Protein TTR misfolding and tissue deposition of amyloid start many years before the appearance of the first symptoms in ATTRv (Arbustini and Merlini, 2014; Adams et al., 2019). This phenomenon leads to the opportunity and the need to identify novel biomarkers to detect earlier phases of the disease and to permit prompter disease treatment. Since several studies have reported the presence of inflammation in ATTRv nerve biopsies, some inflammatory markers have been recently investigated in serum. TNF-α, IFN-β, IL-1β, IL-8, IL-10, and IL-33 were found increased in patients versus healthy controls, but high levels of IL-33, IL-1β, and IL-10 were already seen in stage 0 asymptomatic carriers. Although it is unclear whether such a pattern of inflammatory cytokines is primarily related to the disease pathogenesis or is a secondary effect of tissue damage or to circulating oligomers, it confirms that the body is reacting to the disease much before amyloid deposition or tissue damage take place (Azevedo et al., 2019).

Circulating miRNAs have been investigated in patients with ATTRv and wild type ATTR (ATTRwt) cardiomyopathy. Validation experiments led to the identification of up-regulated miR-339-3p only in ATTRwt but not in patients with heart failure of other origin or with ATTRv, supporting it as a potential candidate biomarker for ATTRwt (Derda et al., 2018). Our pilot study was performed with the main aim to distinguish miRNAs profile between stage 0 and stage 1–2 in subjects carrying a TTR mutation. We found many candidates deregulated in ATTRv patients versus healthy controls, versus CMT patients and versus asymptomatic carriers of TTRv. Our attention was drawn on 12 miRNAs significantly up-regulated with a fold increase higher than 100 in ATTRv patients versus asymptomatic TTRv carriers. Among them, we focused on miR-150-5p and aimed to validate our results and to look for possible pathomechanisms.

MiR-150-5p is expressed in many tissues, with greater distribution in pancreas, lymph nodes, spleen, vascular system, respiratory system, prostate, esophagus, kidney, thyroid, and also in peripheral nerves and myocardium (Ludwig et al., 2016). It regulates various immune cell functions via apoptosis, survival, and proliferation, controlling immune response and inflammatory cytokines, and also plays an important role in the pathogenesis of both solid and hematological cancers (Wu et al., 2010; Jiang et al., 2012; Sang et al., 2016). No data are so far available in the literature about miR-150-5p expression either in different components of normal nerves, i.e., the axon-myelin unit, or in nerves and heart from ATTRv patients or animal models. The miR-150-5p up-regulation found in this study was tested in a larger separate validation cohort and ROC analysis allowed to confirm the discrimination ability of miR-150-5p to differentiate ATTRv patients from asymptomatic carriers of TTRv, with high sensitivity and specificity. Some studies have investigated the different ability of laboratory and instrumental tests to detect early heart involvement and predict clinical worsening in subjects carrying a TTRv but yet completely asymptomatic who have been serially followed-up (Glaudemans et al., 2014; Hutt et al., 2017; Minutoli et al., 2019). Similarly, the discovery of a non-invasive sensitive biomarker specific for ATTRv patients, as supported by present results, needs now to be further validated longitudinally in a large cohort of asymptomatic carriers of TTRv to identify the time of increased expression of serum miR-150-5p and to verify its possible capability to anticipate the appearance of clinical symptoms. If the latter will be confirmed, this could be a strong argument in favor of an early start of now available innovative treatments (Vita et al., 2019) in still asymptomatic carriers.

How increased expression of miR-150-5p could play a pathophysiological role in ATTRv is lacking in the scientific literature, although some findings have been reported regarding other neurodegenerative and cardiac diseases. Decreased plasma miR-150-5p levels have been found in patients with Alzheimer disease (AD) versus healthy controls (Lugli et al., 2015). Increased levels were observed in hippocampal neurons of prion-infected mice and in synaptoneurosomes from prion-infected mouse forebrain, both at terminal stage of the disease (Majer et al., 2012; Boese et al., 2016). Evidences were provided that increased miR-150 levels found in Purkinje neurons of the mouse model of spinocerebellar ataxia type 1 may modulate disease pathogenesis by targeting the expression of RGS8 and VEGFA genes (Rodriguez-Lebron et al., 2013). Moreover, circulating miR-150 was up-regulated in workers with carbon disulfide neuropathy (Guo et al., 2015), and miR-150-5p down-regulated in patients with advanced heart failure (Scrutinio et al., 2017). So, we decided to investigate the possible crosstalk between miR-150-5p and three different factors, CREB, BDNF, and NGF, whose roles in central and peripheral nervous systems and in the heart are known, using a cell culture model. Human primary SCs cultures were chosen as an easy model on the evidence of miR-150-5p expression in nerves (Ludwig et al., 2016). Although peripheral nerve involvement occurring in ATTRv is mainly an axonal type of neuropathy, SCs are known to participate with an active role during axonal degeneration, although through still poorly understood molecular and cellular mechanisms (Wong et al., 2017).

CREB has a multifaceted role in the nervous system, mostly in neurodevelopment, synaptic plasticity, and neuroprotection (Sakamoto et al., 2011). Recent evidences have been presented supporting the possibility that a dysregulation of CREB signaling is associated to cerebral amyloidosis, formation of tangle-like structures and microglial clustering, and cognitive decline in AD and its animal model (Kempf et al., 2016; Bartolotti and Lazarov, 2019). Moreover, CREB protein family of transcription factors is involved in cardiac growth, ventricular remodeling, and heart failure (Kobrinsky et al., 2011; Kreusser and Backs, 2014; Zhou et al., 2018).

BDNF plays a key role in neurogenesis and synaptic repair and is implicated in numerous neurodegenerative disorders (Bawari et al., 2019). Low plasma BDNF levels have been found significantly associated with brain amyloid burden measured with Pittsburg Compound B in AD and mild cognitive impairment patients, supporting a pathogenic and a peripheral signature role of BDNF (Hwang et al., 2015). Equally BDNF is a focal growth factor which regulates the response of cardiovascular system to acute and chronic injury, modulating contractility, neoangiogenesis, apoptosis and survival of cardiac myocytes, vascular muscle cells, and endothelial cells (Kermani and Hempstead, 2019).

Finally, NGF is the firstly discovered and best characterized neurotrophic factor, able to stimulate neuronal growth and differentiation in central, peripheral, and sympathetic nervous systems (Freed, 1976; Aloe et al., 2015). Likewise, NGF plays a role in cardiac physiopathology. Its levels increase following myocardial injury, most likely leading to sympathetic nerve sprouting, but decrease when heart failure develops, an event that may participate to defective innervation and cardiac failure (Govoni et al., 2011).

MiRNAs are important post-transcriptional regulators which participate to axonal guidance in the central nervous system, and to proliferation and migration of SCs and axonal growth in the peripheral nervous system (Wang et al., 2019). Our SCs culture model allowed to demonstrate for the first time that miR-150-5p is a powerful negative regulator of CREB gene, BDNF gene, and, to a lesser extent, NGF gene expression, providing an additional basis for further investigations of their role in neurodegeneration and specifically in ATTRv pathogenesis.

This pilot study has some limits. One is the small sample size, but we have to consider that ATTRv amyloidosis is a rare disease and an exclusion criterion was the presence of concomitant major disease. Moreover, 12 miRNAs were found up-regulated with a fold increase higher than 100 in ATTRv patients versus asymptomatic TTRv carriers. However, we concentrated on one of them, miR-150-5p, in this study, with the aim to validate it and to look for associated pathophysiology. Nevertheless, all the others miRNAs have a translational potential and may point to future directions in the analysis of this emerging class of biomarkers. Furthermore, the biological basis of our observations in SCs culture needs to be confirmed in other experimental models.

In conclusion, identification of dysregulated miRNAs can help in understanding the complex pathomechamism underlying the development of ATTRv and related multisystemic pathology. The current results showed that some miRNAs are up-regulated and other down-regulated in stage 1-2 ATTRv patients versus stage 0 subjects. Serum level of miR-150-5p were able to well discriminate stage 1-2 versus stage 0. Our SCs culture model demonstrated that miR-150-5p may modulate the expression of CREB, BDNF and NGF genes, supporting their role in ATTRv pathogenesis. Further investigations on the role of circulating miR-150-5p to predict the shift of TTRv carriers from stage 0 to stage 1 are needed. Moreover, another future line of investigation should examine whether miR-150-5p circulating levels are responsive to the innovative treatments now available.

## Data Availability Statement

The datasets generated for this study are available on request to the corresponding author.

## Ethics Statement

The study was approved by Comitato Etico di Messina, AOU Policlinico “G. Martino”, Messina, Italy. The patients/participants provided their written informed consent to participate in this study.

## Author Contributions

GLV, M’HA, CR, RD, AT, GV, and AM contributed to the conception and design of the study. GLV, M’HA, FP, RO, MRu, LG, CB, MRa, RD, GV, and AM contributed to the acquisition and analysis of data. GLV, M’HA, FP, RO, MRu, LG, CB, MRa, CR, RD, AT, GV, and AM contributed to drafting the text and preparing the figures. All authors approved the final version.

## Conflict of Interest

The authors declare that the research was conducted in the absence of any commercial or financial relationships that could be construed as a potential conflict of interest.

## Footnotes

1 http://www.microrna.gr/tarbase

2 http://snf-515788.vm.okeanos.grnet.gr/

3 http://www.tm4.org

## Supplementary Material

List of miRNAs expressed in the single subjects has been deposited in NCBI’s Gene Expression Omnibus and is accessible through GEO Series accession number GSE149665 (https://www.ncbi.nlm.nih.gov/geo/query/acc.cgi?acc~=~GSE149665).

TABLE S1 Data from validation set.

TABLE S2 Data from Schwann cells culture experiments.

## References

[B1] Adams D. Cauquil C. Labeyrie C. (2017). Familial amyloid polyneuropathy. *Curr. Opin. Neurol.* 30 481–489. 10.1097/WCO.0000000000000476 28678039

[B2] Adams D. Koike H. Slama M. Coelho T. (2019). Hereditary transthyretin amyloidosis: a model of medical progress for a fatal disease. *Nat. Rev. Neurol.* 15 387–404. 10.1038/s41582-019-0210-4 31209302

[B3] Adams D. Suhr O. B. Hund E. Obici L. Tournev I. Campistol J. M. (2016). First European consensus for diagnosis, management, and treatment of transthyretin familial amyloid polyneuropathy. *Curr. Opin. Neurol.* 29(Suppl. 1), S14–S26. 10.1097/WCO.0000000000000289 26734952 PMC4739312

[B4] Aloe L. Rocco M. L. Balzamino B. O. Micera A. (2015). Nerve growth factor: a focus on neuroscience and therapy. *Curr. Neuropharmacol.* 13 294–303. 10.2174/1570159x13666150403231920 26411962 PMC4812798

[B5] Arbustini E. Merlini G. (2014). Early identification of transthyretin-related hereditary cardiac amyloidosis. *JACC Cardiovasc. Imaging* 7 511–514. 10.1016/j.jcmg.2014.03.007 24831211

[B6] Azevedo E. P. Guimaraes-Costa A. B. Bandeira-Melo C. Chimelli L. Waddington-Cruz M. Saraiva E. M. (2019). Inflammatory profiling of patients with familial amyloid polyneuropathy. *BMC Neurol.* 19:146. 10.1186/s12883-019-1369-4 31253122 PMC6599258

[B7] Baltimore D. Boldin M. P. O’Connell R. M. Rao D. S. Taganov K. D. (2008). MicroRNAs: new regulators of immune cell development and function. *Nat. Immunol.* 9 839–845. 10.1038/ni.f.209 18645592

[B8] Bartolotti N. Lazarov O. (2019). CREB signals as PBMC-based biomarkers of cognitive dysfunction: a novel perspective of the brain-immune axis. *Brain Behav. Immun.* 78 9–20. 10.1016/j.bbi.2019.01.004 30641141 PMC6488430

[B9] Bawari S. Tewari D. Argüelles S. Sah A. N. Nabavi S. F. Xu S. (2019). Targeting BDNF signaling by natural products: novel synaptic repair therapeutics for neurodegeneration and behavior disorders. *Pharmacol. Res.* 148:104458. 10.1016/j.phrs.2019.104458 31546015

[B10] Boese A. S. Saba R. Campbell K. Majer A. Medina S. Burton L. (2016). MicroRNA abundance is altered in synaptoneurosomes during prion disease. *Mol. Cell. Neurosci.* 71 13–24. 10.1016/j.mcn.2015.12.001 26658803

[B11] Derda A. A. Pfanne A. Bär C. Schimmel K. Kennel P. J. Xiao K. (2018). Blood-based microRNA profiling in patients with cardiac amyloidosis. *PLoS One* 13:e0204235. 10.1371/journal.pone.0204235 30332417 PMC6192556

[B12] Di Pietro V. Porto E. Ragusa M. Barbagallo C. Davies D. Forcione M. (2018). Salivary microRNAs: diagnostic markers of mild traumatic brain injury in contact-sport. *Front. Mol. Neurosci.* 11:290. 10.3389/fnmol.2018.00290 30177873 PMC6109773

[B13] Fattahi M. Eskandari N. Sotoodehnejadnematalahi F. Shaygannejad V. Kazemi M. (2020). Comparison of the expression of miR-326 between interferon beta responders and non-responders in relapsing-remitting multiple sclerosis. *Cell J.* 22 92–95. 10.22074/cellj.2020.6486 31606972 PMC6791062

[B14] Freed W. J. (1976). The role of nerve-growth factor (NGF) in the central nervous system. *Brain Res. Bull.* 1 393–412. 10.1016/0361-9230(76)90033-2 61795

[B15] Glaudemans A. W. van Rheenen R. W. van den Berg M. P. Noordzij W. Koole M. Blokzijl H. (2014). Bone scintigraphy with (99m)technetium-hydroxymethylene diphosphonate allows early diagnosis of cardiac involvement in patients with transthyretin-derived systemic amyloidosis. *Amyloid* 21 35–44. 10.3109/13506129.2013.871250 24455993

[B16] Govoni S. Pascale A. Amadio M. Calvillo L. D’Elia E. Cereda C. (2011). NGF and heart: is there a role in heart disease? *Pharmacol. Res.* 63 266–277. 10.1016/j.phrs.2010.12.017 21195180

[B17] Guo L. Luo C. Fan J. Hou Z. Ji X. Chen F. (2015). Serum miRNA profiling identifies miR-150/30a as potential biomarker for workers with damaged nerve fibers from carbon disulfide. *Ind. Health* 53 38–47. 10.2486/indhealth.2014-0120 25224332 PMC4331193

[B18] Huang Z. Zhang L. Zhu D. Shan X. Zhou X. Qi L. W. (2017). A novel serum microRNA signature to screen esophageal squamous cell carcinoma. *Cancer Med.* 6 109–119. 10.1002/cam4.973 28035762 PMC5269712

[B19] Hutt D. F. Fontana M. Burniston M. Quigley A. M. Petrie A. Ross J. C. (2017). Prognostic utility of the Perugini grading of 99mTc-DPD scintigraphy in transthyretin (ATTR) amyloidosis and its relationship with skeletal muscle and soft tissue amyloid. *Eur. Heart J. Cardiovasc. Imaging* 18 1344–1350. 10.1093/ehjci/jew325 28159995

[B20] Hwang K. S. Lazaris A. S. Eastman J. A. Teng E. Thompson P. M. Gylys K. H. (2015). Plasma BDNF levels associate with Pittsburgh compound B binding in the brain. *Alzheimers Dement.* 1 187–193. 10.1016/j.dadm.2015.01.005 26207261 PMC4507280

[B21] Jiang X. Huang H. Li Z. Li Y. Wang X. Gurbuxani S. (2012). Blockade of miR-150 maturation by MLL-fusion/MYC/LIN-28 is required for MLL-associated leukemia. *Cancer Cell* 22 524–535. 10.1016/j.ccr.2012.08.028 23079661 PMC3480215

[B22] Jonker D. L. Hazenberg B. P. C. Nienhuis H. L. A. Slart R. H. J. A. Glaudemans A. W. J. M. Noordzij W. (2018). Imaging cardiac innervation in hereditary transthyretin (ATTRm) amyloidosis: a marker for neuropathy or cardiomyopathy in case of heart failure? *J. Nucl. Cardiol.* [Epub ahead of print]. 10.1007/s12350-018-01477-y 30374850 PMC7599160

[B23] Kempf S. J. Metaxas A. Ibáñez-Vea M. Darvesh S. Finsen B. Larsen M. R. (2016). An integrated proteomics approach shows synaptic plasticity changes in an APP/PS1 Alzheimer’s mouse model. *Oncotarget* 7 33627–33648. 10.18632/oncotarget.9092 27144524 PMC5085108

[B24] Kermani P. Hempstead B. (2019). BDNF actions in the cardiovascular system: roles in development, adulthood and response to injury. *Front. Physiol.* 10:455. 10.3389/fphys.2019.00455 31105581 PMC6498408

[B25] Kobrinsky E. Duong S. Q. Sheydina A. Soldatov N. M. (2011). Microdomain organization and frequency-dependence of CREB-dependent transcriptional signaling in heart cells. *FASEB J.* 25 1544–1555. 10.1096/fj.10-176198 21248242 PMC3079295

[B26] Kong Y. W. Ferland-McCollough D. Jackson T. J. Bushell M. (2012). microRNAs in cancer management. *Lancet Oncol.* 13 e249–e258. 10.1016/S1470-2045(12)70073-6 22652233

[B27] Kreusser M. M. Backs J. (2014). Integrated mechanisms of CaMKII-dependent ventricular remodeling. *Front. Pharmacol.* 5:36. 10.3389/fphar.2014.00036 24659967 PMC3950490

[B28] Li X. Teng C. Ma J. Fu N. Wang L. Wen J. (2019). miR-19 family: a promising biomarker and therapeutic target in heart, vessels and neurons. *Life Sci.* 232:116651. 10.1016/j.lfs.2019.116651 31302195

[B29] Ludwig N. Leidinger P. Becker K. Backes C. Fehlmann T. Pallasch C. (2016). Distribution of miRNA expression across human tissues. *Nucleic Acids Res.* 44 3865–3877. 10.1093/nar/gkw116 26921406 PMC4856985

[B30] Lugli G. Cohen A. M. Bennett D. A. Shah R. C. Fields C. J. Hernandez A. G. (2015). Plasma exosomal miRNAs in persons with and without Alzheimer disease: altered expression and prospects for biomarkers. *PLoS One* 10:e0139233. 10.1371/journal.pone.0139233 26426747 PMC4591334

[B31] Luigetti M. Bisogni G. Romano A. Di Paolantonio A. Barbato F. Primicerio G. (2018). Sudoscan in the evaluation and follow-up of patients and carriers with TTR mutations: experience from an Italian Centre. *Amyloid* 25 242–246. 10.1080/13506129.2018.1545640 30638075

[B32] Majer A. Medina S. J. Niu Y. Abrenica B. Manguiat K. J. Frost K. L. (2012). Early mechanisms of pathobiology are revealed by transcriptional temporal dynamics in hippocampal CA1 neurons of prion infected mice. *PLoS Pathog.* 8:e1003002. 10.1371/journal.ppat.1003002 23144617 PMC3493483

[B33] Mazzeo A. Russo M. Di Bella G. Minutoli F. Stancanelli C. Gentile L. (2015). Transthyretin-related familial amyloid polyneuropathy (TTR-FAP): a single-center experience in Sicily, an Italian endemic area. *J. Neuromuscul. Dis.* 2 S39–S48. 10.3233/JND-150091 27858761 PMC5271421

[B34] Minutoli F. Di Bella G. Mazzeo A. Laudicella R. Gentile L. Russo M. (2019). Serial scanning with 99mTc-3, 3-diphosphono-1, 2-propanodicarboxylic acid (99mTc-DPD) for early detection of cardiac amyloid deposition and prediction of clinical worsening in subjects carrying a transthyretin gene mutation. *J. Nucl. Cardiol.* [Epub ahead of print]. 10.1007/s12350-019-01950-2 31741327

[B35] Molasy M. Walczak A. Szaflik J. Szaflik J. P. Majsterek I. (2017). MicroRNAs in glaucoma and neurodegenerative diseases. *J. Hum. Genet.* 62 105–112. 10.1038/jhg.2016.91 27412874

[B36] Ng R. Song G. Roll G. R. Frandsen N. M. Willenbring H. (2012). A microRNA-21 surge facilitates rapid cyclin D1 translation and cell cycle progression in mouse liver regeneration. *J. Clin. Invest.* 122 1097–1108. 10.1172/JCI46039 22326957 PMC3287214

[B37] Obici L. Kuks J. B. Buades J. Adams D. Suhr O. B. Coelho T. (2016). Recommendations for presymptomatic genetic testing and management of individuals at risk for hereditary transthyretin amyloidosis. *Curr. Opin. Neurol.* 29(Suppl. 1), S27–S35. 10.1097/WCO.0000000000000290 26734953 PMC4739313

[B38] Png K. J. Halberg N. Yoshida M. Tavazoie S. F. (2011). A microRNA regulon that mediates endothelial recruitment and metastasis by cancer cells. *Nature* 481 190–194. 10.1038/nature10661 22170610

[B39] Rayner K. J. Esau C. C. Hussain F. N. McDaniel A. L. Marshall S. M. van Gils J. M. (2011). Inhibition of miR-33a/b in non-human primates raises plasma HDL and lowers VLDL triglycerides. *Nature* 478 404–407. 10.1038/nature10486 22012398 PMC3235584

[B40] Rodriguez-Lebron E. Liu G. Keiser M. Behlke M. A. Davidson B. L. (2013). Altered Purkinje cell miRNA expression and SCA1 pathogenesis. *Neurobiol. Dis.* 54 456–463. 10.1016/j.nbd.2013.01.019 23376683 PMC3629010

[B41] Sakamoto K. Karelina K. Obrietan K. (2011). CREB: a multifaceted regulator of neuronal plasticity and protection. *J. Neurochem.* 116 1–9. 10.1111/j.1471-4159.2010.07080.x 21044077 PMC3575743

[B42] Sang W. Wang Y. Zhang C. Zhang D. Sun C. Niu M. (2016). MiR-150 impairs inflammatory cytokine production by targeting ARRB-2 after blocking CD28/B7 costimulatory pathway. *Immunol. Lett.* 172 1–10. 10.1016/j.imlet.2015.11.001 26549736 PMC4846526

[B43] Scrutinio D. Conserva F. Passantino A. Iacoviello M. Lagioia R. Gesualdo L. (2017). Circulating microRNA-150-5p as a novel biomarker for advanced heart failure: a genome-wide prospective study. *J. Heart Lung Transplant.* 36 616–624. 10.1016/j.healun.2017.02.008 28259597

[B44] Vita G. Vita G. L. Stancanelli C. Gentile L. Russo M. Mazzeo A. (2019). Genetic neuromuscular disorders: living the era of a therapeutic revolution. Part 1: peripheral neuropathies. *Neurol. Sci.* 40 661–669. 10.1007/s10072-019-03778-7 30847674

[B45] Vita G. L. Polito F. Oteri R. Arrigo R. Ciranni A. M. Musumeci O. (2018). Hippo signaling pathway is altered in Duchenne muscular dystrophy. *PLoS One* 13:e0205514. 10.1371/journal.pone.0205514 30304034 PMC6179272

[B46] Wang X. Chen Q. Yi S. Liu Q. Zhang R. Wang P. (2019). The microRNAs let-7 and miR-9 down-regulate the axon-guidance genes Ntn1 and Dcc during peripheral nerve regeneration. *J. Biol. Chem.* 294 3489–3500. 10.1074/jbc.RA119.007389 30626732 PMC6416429

[B47] Wong K. M. Babetto E. Beirowski B. (2017). Axon degeneration: make the Schwann cell great again. *Neural Regen. Res.* 12 518–524. 10.4103/1673-5374.205000 28553320 PMC5436338

[B48] Wu Q. Jin H. Yang Z. Luo G. Lu Y. Li K. (2010). MiR-150 promotes gastric cancer proliferation by negatively regulating the pro-apoptotic gene EGR2. *Biochem. Biophys. Res. Commun.* 392 340–345. 10.1016/j.bbrc.2009.12.182 20067763

[B49] Zhou H. Li N. Yuan Y. Jin Y. G. Guo H. Deng W. (2018). Activating transcription factor 3 in cardiovascular diseases: a potential therapeutic target. *Basic Res. Cardiol.* 113:37. 10.1007/s00395-018-0698-6 30094473

[B50] Zouari H. G. Ng Wing Tin S. Wahab A. Damy T. Lefaucheur J. P. (2019). Assessment of autonomic innervation of the foot in familial amyloid polyneuropathy. *Eur. J. Neurol.* 26:94-e10. 10.1111/ene.13774 30102818

