# Supplementary material for: Circulating microRNAs Profile in Patients With Transthyretin Variant Amyloidosis
Source: Front Mol Neurosci. 2020 Jun 23;13:102. doi: 10.3389/fnmol.2020.00102 (PMC7325132; doi:10.3389/fnmol.2020.00102)
Supplement: TABLE S1 — Data from validation set. [file Table_1.docx]

Average of the relative quantity, obtained by triplicate assays, of miR-150-5p by RT-qPCR in ATTRv patients vs asymptomatic TTRv carriers (validation set).

| ATTRv patients | | Asymptomatic TTRv carriers | |
| --- | --- | --- | --- |
| #1 | 4.01 | #1 | 2.01 |
| #2 | 3.21 | #2 | 2.01 |
| #3 | 3.24 | #3 | 2.21 |
| #4 | 2.98 | #4 | 1.97 |
| #5 | 2.22 | #5 | 3.54 |
| #6 | 3.54 | #6 | 2.01 |
| #7 | 4.12 | #7 | 1.89 |
| #8 | 4.32 | #8 | 2.32 |
| #9 | 2.47 | #9 | 2.21 |
| #10 | 2.78 | #10 | 2.09 |
| #11 | 4.02 | #11 | 2.17 |
| #12 | 3.56 | #12 | 2.02 |
| #13 | 4.52 | #13 | 1.95 |
| #14 | 4.05 | #14 | 2.05 |
| #15 | 3.65 | #15 | 2.11 |
| #16 | 4.02 | #16 | 2.23 |
| #17 | 4.11 | #17 | 2.21 |
| #18 | 3.41 | #18 | 2.12 |
| #19 | 3.27 | #19 | 1.97 |
| #20 | 3.26 | #20 | 2.22 |
| #21 | 3.12 | #21 | 2.31 |
| #22 | 4.57 | #22 | 1.96 |
| #23 | 3.87 | #23 | 2.03 |
| #24 | 3.12 |  |  |
