# Supplementary material for: Circulating microRNAs Profile in Patients With Transthyretin Variant Amyloidosis
Source: Front Mol Neurosci. 2020 Jun 23;13:102. doi: 10.3389/fnmol.2020.00102 (PMC7325132; doi:10.3389/fnmol.2020.00102)
Supplement: TABLE S2 — Data from Schwann cells culture experiments. [file Table_2.DOCX]

**Effect of transfection of miR-150-5p mimic/inhibitor into human primary Schwann cells on miR-150-5p expression (average of results obtained by triplicate assays).**

| **Ctrl** | **miR-150-5p mimic** | **miR-150-5p inhibitor** | **Ctrl + vector** |
| --- | --- | --- | --- |
| 0.1 | 3.4 | -0.36 | 0.3 |
| 0.11 | 4.1 | -0.28 | 0.2 |
| 0.09 | 3.8 | -0.26 | 0.85 |
| 0.12 | 3.9 | -0.15 | 0.75 |
| 0.11 | 2.9 | -0.26 | 0.12 |
| 0.095 | 3.5 | -0.34 | 0.29 |
| 0.096 | 4.3 | -0.3 | 0.9 |
| 0.1 | 4.2 | -0.18 | 0.4 |
| 0.12 | 4.1 | -0.15 | 0.7 |

**Effect of transfection of miR-150-5p mimic/inhibitor into human primary Schwann cells on CREB protein (A) and mRNA (B) level (average of results obtained by triplicate assays).**

**A**

| **Ctrl** | **miR-150-5p mimic** | **miR-150-5p inhibitor** |
| --- | --- | --- |
| 5.2 | 0.9 | 5 |
| 3.2 | 0.5 | 2.5 |
| 3.6 | 0.9 | 5.6 |
| 4 | 1 | 4.9 |
| 4.4 | 0.4 | 6 |
| 5.5 | 0.84 | 4.9 |
| 4.6 | 0.7 | 4.5 |
| 2.3 | 0.39 | 2.38 |
| 4.9 | 0.8 | 5.8 |

**B**

| **Ctrl** | **miR-150-5p mimic** | **miR-150-5p inhibitor** |
| --- | --- | --- |
| 5 | 0.71 | 5.4 |
| 4.7 | 1 | 2.68 |
| 2.9 | 1.2 | 4.7 |
| 5.5 | 0.9 | 5.5 |
| 4.7 | 0.8 | 3 |
| 2.75 | 0.58 | 5.9 |
| 2.6 | 1.3 | 2.9 |
| 3.2 | 1.43 | 3.45 |
| 4.2 | 1.3 | 5.4 |

**Effect of transfection of miR-150-5p mimic/inhibitor into human primary Schwann cells on BDNF protein (A) and mRNA (B) level (average of results obtained by triplicate assays).**

**A**

| **Ctrl** | **miR-150-5p mimic** | **miR-150-5p inhibitor** |
| --- | --- | --- |
| 7.3 | 2.2 | 3.5 |
| 6.8 | 1.8 | 5 |
| 5.8 | 1.9 | 6.5 |
| 4 | 1.6 | 3.8 |
| 6.8 | 2.1 | 6.7 |
| 6.4 | 1.5 | 5.6 |
| 6.3 | 0.9 | 5.5 |
| 4 | 1.1 | 3.4 |
| 7.6 | 2 | 5 |

**B**

| **Ctrl** | **miR-150-5p mimic** | **miR-150-5p inhibitor** |
| --- | --- | --- |
| 5.8 | 1.9 | 5.6 |
| 4.8 | 1.6 | 3.4 |
| 6 | 0.6 | 6.2 |
| 5.9 | 1.7 | 4.7 |
| 3.2 | 0.9 | 3.6 |
| 6.1 | 0.8 | 3.3 |
| 6.3 | 2.3 | 5.3 |
| 5.5 | 1.9 | 5.6 |
| 3.2 | 0.9 | 5.7 |

**Effect of transfection of miR-150-5p mimic/inhibitor into human primary Schwann cells on NGF protein (A) and mRNA (B) level (average of results obtained by triplicate assays).**

**A**

| **Ctrl** | **miR-150-5p mimic** | **miR-150-5p inhibitor** |
| --- | --- | --- |
| 2.6 | 1.2 | 5.4 |
| 3.4 | 1.5 | 2.8 |
| 4.6 | 1.8 | 3.9 |
| 4.8 | 2.5 | 2.2 |
| 5.6 | 1.7 | 5.4 |
| 5.3 | 2.1 | 2.9 |
| 4.6 | 1.4 | 4.6 |
| 2.8 | 1.6 | 5.2 |
| 2.5 | 2.4 | 5.5 |

**B**

| **Ctrl** | **miR-150-5p mimic** | **miR-150-5p inhibitor** |
| --- | --- | --- |
| 5.7 | 2.5 | 4.9 |
| 5.2 | 1.6 | 5.3 |
| 4.6 | 2.4 | 5.5 |
| 2.8 | 2.2 | 3.2 |
| 3.1 | 2.3 | 2.9 |
| 5.3 | 2.4 | 5.4 |
| 5.5 | 1.8 | 4.8 |
| 5.4 | 1.4 | 4.9 |
| 2.9 | 2.4 | 1.9 |
